# Development of a Valid and Reliable Diabetes Empowerment Scale: An Iranian Version

**Published:** 2012-05-30

**Authors:** A Tol, G R Sharifirad, A G Pourreza, A Rahimi, D Shojaeezadeh, M R Mohajeritehrani, F Alhani

**Affiliations:** 1 Department of Health Education and Promotion, School of Public Health, Isfahan University of Medical Sciences, Isfahan, Iran; 2 Department of Health Management and Economics, School of Public Health, Tehran University of Medical Sciences, Tehran, Iran; 3 Department of Epidemiology and Biostatistics, School of Public Health, Tehran University of Medical Sciences, Tehran, Iran; 4 Endocrine and Metabolism Research Centre, Tehran University of Medical Sciences, Tehran, Iran; 5 School of Medicine, Tarbiat Modarres University, Tehran, Iran

**Keywords:** Empowerment, Type 2 diabetes, Reliability, Validity, Iran

## Abstract

**Background:**

Patient empowerment can enhance the outcomes of care such as metabolic control as well as quality of their life. This study evaluates the Iranian version for development of a valid and reliable diabetes empowerment scale.

**Methods:**

Validity and reliability of Iranian version of Diabetes Empowerment Scale (DES-LF) were measured through a cross-sectional study. DES-LF was evaluated through a qualitative and quantitative study by 160 type 2 diabetic patients.

**Results:**

Reliability and validity of the scale and its 3 subscales, namely, managing the psychosocial aspects of diabetes (α=0.94), assessing dissatisfaction and readiness to change (α=0.96), and setting and achieving diabetes goals (α=0.96) were approved by a psychometric analysis.

**Conclusion:**

Findings approved the reliability and validity of the Iranian version of DES-LF for patient education and psychosocial interventions among Iranian people with type 2 diabetes.

## Introduction

Type 2 diabetes is one of the most health concerns in worldwide.[1] It is estimated that a 54% increase will occur in adult diabetic patients from 2010 to 2030.[2] In fact, type 2 diabetes has a strong position among epidemiologic transition diseases which affects people from different ages, sexes, ethnic groups, and social classes.[3] Today, curative and medical approaches towards dealing with patients and disease were mainly replaced by preventive and participatory approaches in which patients played a determinant role for their health.[4]

As a very positive concept, empowerment meets mainly strengths and potential abilities among people in general and patients in particular. Through the empowerment process, emphasis is placed not on weaknesses and problems but on encouragement and abilities of individuals.[5] Empowerment is defined by learning and practicing new concepts and behavior. Empowerment enables individuals to make decision about their health and behave in accordance with their own decision and responsibilities.[6] Empowerment consists of three main categories as knowledge, behavioral skills, and self-responsibility.[4] Somebody believes that diabetic patients should experience self care and direct responsibility of dealing with their illness, because their own decisions develop a great impact on their health.[6]

Studies demonstrated that patient empowerment can enhance the outcomes of care such as metabolic control as well as quality of their life.[7] Making informed decisions for diabetic patients in self- management of their illness through the process of empowerment has been indicated in Anderson et al. studies.[7][8] Empowerment of diabetic patients is measured through a scale, namely (DES) which was developed and tested. Long form (28 items) and short form (8 items) of this scale is repeatedly utilized in several studies.[9]

Because of almost high prevalence of diabetes in Iran,[10][11] and a great need for a valid and reliable instrument for studies as well as policy making in shifting the current medical approach for diabetes with empowerment approach to diabetic patients,[12] validation of the scale developed by Anderson et al. (2000) seems to be a necessity. In this paper the process of validation of the scale is followed.

## Materials and Methods

One hundred and sixty patients provided a minimum of five respondents per item on the F-DES-28 for factor analysis purposes.[13] Systematic random sampling method was employed to select patient from a recorded available list of diabetic patients. As a whole, 160 eligible patients aged ≥44 years were selected from patients during a 6 months period of data collection. Patients were also assured about the confidentiality of personal data, and their right to whether or not to participate in the study process. By providing the patients' consensus for the study, an appointment was set to make a structured interview with patients. Moreover, information about HbA1c results of patients was collected from the last medical record of each patient.

The study design consisted of two qualitative and quantitative stages. In qualitative stage, the translation and back translation of the original version of DES-28 were conducted. Back translation was reviewed by a committee in order to achieve a modified version of the original copy. The translation committee (three researchers and two translators) checked and agreed upon the provided version of F-DES-28 as a comprehensive representative of the original version in terms of wording and content. Two focus group discussions =FGD, (7 persons in each groups) were developed in order to achieve a consensus on the form and content of the translated version. The members of the groups were invited as key informed to evaluate and approve the translated version of FDES-28 as culturally and linguistically appropriate Provided version of F-DES-28 by FGDs was put for content analysis by three independent researchers. The analysis demonstrated that the version provided by FGDs was almost entirely the same as original version in terms of wording and content. The prepared version transferred to the panel of experts (included 6 persons) was responsible for final version of F-DES-28.

This panel included 2 endocrinologists and 4 diabetes educators and was responsible for the assessment of content validity of F-DES-28. The original DES-28 and the focus group F-DES-28 were sent to each member of the panel, who were bilingual. Content validity was assessed by asking the members to rate each item as a valid measure of the construct using a five-point Likert scale (1 _ strongly disagree, 5 _ strongly agree). A content validity ratio was calculated for each item and for the overall, F-DES-28. Content validity ratio was calculated as equal to 4.9 which is higher than acceptable ratio (≥3). In addition, the panel was asked to make comments on individual items in relation to the accuracy clarity, style, and cultural relevance of the translation. Minor changes were suggested (on the fluency of four items), and a panel-modified version was developed. In quantitative stage, the final version was piloted, then necessary corrections were made and post pilot version was prepared. This version was applied for the study population. Although applying this version to a subsample of the study population as a retest was possible, we tried to retest it through the whole sample of the study. As a result, the validity and reliability were achieved.

The patients in the pilot study initially commented on the non specificity of the wording in some of the items involving “setting goals”. Some patients expected more clarification about some concepts such as “setting goal” in their daily living activists. More explanations were made to the patients. It helped the patients that found it easy to respond to these nonspecific items. A post pilot version of the scale was developed. The collected data were analyzed by using SPSS software (Version 11.5, Chicago, IL, USA). The significance level was set at p0.01.

## Results

The response rate was 75%. The majority aged between 45 and 54 years (48.67±7.99 years) and half of them were diagnosed as diabetic patients during the past 6 years (5.62±3.81 years), and 59% demonstrated borderline metabolic control according to World Health Organization criteria (7.72±1.30).[14] Table 1 demonstrates the demographic and clinical data of the participants. "Managing psychosocial aspects of diabetes" demonstrated as the most important domain among domains analyzed by factor analysis. Eigen value for this domain was 2.54 which was reflected in Table 2. This table also shows descriptive statistics for the subscales. Reliability of F-DES-28 scale was measured by test-retest and resulted in α choronbach of 0.976 (95% CI 0.970-0.981). The rate of α chronbach among the subscales ranged from 0.941 to 0.968. A significant correlation was found between global scale and HbA1c among the respondents which indicated that the higher the F-DES-28 scores, the lower the HbA1c values (r=-0.75, p˂0.001). After controlling the effects of age, educational level, and duration of living with diabetes, the correlation between global empowerment scale and HbA1c of type 2 diabetic respondents remained significant (r=-0.75, p˂0.001).

**Table 1:** Demographic and clinical data (N=160)

**Frequency**	**(%)**
Age(Years)	
≤44	54(33.7)
45-54	74(46.3)
≥55	32(20)
Sex	
Male	79(49.4)
Female	81(50.6)
Level of education	
Up to diploma	89(55.6)
Diploma and higher	71(44.4)
Marital Status	
Married	140(87.5)
Unmarried	20(12.5)
Length of time since diagnosed( Years)	
≤ 2	39(24.4)
3-5	53(33.2)
≥6	68(43.4)
Metabolic control(HbA1C)	
Optimal control (< 7.0%)	42(25.5)
Borderline control (7.0- 8.5%)	86(59.1)
Poor Control (> 8.5%)	24(15.4)

**Table 2:** Descriptive statistics for F-DES-28

**Domain**	**Number of items**	**Means±SD** **(Range)**	**Eigen** **Value**	**Variance ** ** (%)**
**Managing the psychosocial aspect of diabetes **	9	3.01±0.91 (1-4.33)	2.54	84.70
**Assessing dissatisfaction and ** **readiness to change **	9	3.22±1.15 (1.11- 4.89)	0.299	9.97
**Setting and achieving diabetes goal **	10	3.19±1.17 (1-4.90)	0.159	5.31
**Total**	28	3.14±1.00 (1.21-4.61)	-	-

## Discussion

Findings of the study provided the support for the construct validity and test-retest reliability of the FDES-28. The coefficient for the three subscales and the global F-DES-28 was satisfactory. The test-retest reliability of the F-DES-28 was supported by α chronbach of the subsample of 160 patients when tested after a period of 2 weeks (α=0.977). Spearman coefficient was calculated for all 28 items of the scale and results demonstrated Spearman coefficient more than 0.6. Item 11 was an exception in this respect. In order to determine its effect on the whole scale, reliability α chronbach calculated two times: once for the whole 28 items and the other for 27 items. Resulted α chronbach for these was 0.977 and 0.976 respectively which indicates no significant effect for item 11 with Spearman coefficient of 0.49. Though the application of the scale is easy, its generalization will depend on the size of the sample and population included in the study. In order to estimate empowerment in type 2 diabetic patients, it is necessary to have a valid and reliable scale that provides real information. F-DES-28 can be helpful to determine global empowerment score and related domains to provide more appropriate, relevant teaching materials and to tailor suitable health promotion interventions in type 2 diabetic patients.
